# Supplementary material for: Study of the Counter Cation Effects on the Supramolecular Structure and Electronic Properties of a Dianionic Oxamate-Based {NiII2} Helicate
Source: Molecules. 2023 Feb 23;28(5):2086. doi: 10.3390/molecules28052086 (PMC10003781; doi:10.3390/molecules28052086)
Supplement: Supplementary file 1 [file molecules-28-02086-s001.zip › molecules-2218918-supplementary.pdf]

molecules

MDPI

Article

Study of the Counter Cation Effects on the Supramolecular Structure and Electronic Properties of a Dianionic Oxamate-Based $\{Ni^{II}_2\}$ Helicate

Cintia A. Simosono [1], Rafaela M. R. da Silva [1], Nathália R. De Campos [1], Marye Agnes R. Silva [1], Antônio C. Doriguetto [1], Leonã S. Flores [2], Charlane C. Correa [2], Tatiana R. G. Simões [3], Ana Karoline S. M. Valdo [4], Felipe T. Martins [4], Flávio Garcia [5], Guilherme P. Guedes [6], Breno R. L. Galvão [7], Juliana Cancino-Bernardi [8], Ricardo D. dos Reis [9], Humberto O. Stumpf [10], Danielle D. Justino [7], Paulo F. R. Ortega [7], Walace D. do Pim [7], Miguel Julve [11,*] and Maria Vanda Marinho [1,*]

1 Instituto de Química, Universidade Federal de Alfenas, Campus Santa Clara, Alfenas 37133-840, MG, Brazil; cintiasimosono@hotmail.com (C.A.S.); rafaela.silva@sou.unifal-mg.edu.br (R.M.R.d.S.); nathaliarodriguescampos@yahoo.com.br (N.R.D.C.); marye.silva@sou.unifal-mg.edu.br (M.A.R.S.); doriguetto@gmail.com (A.C.D.)

2 Departamento de Química, Universidade Federal de Juiz de Fora, Campus Martelos, Juiz de Jora 36036-900, MG, Brazil; lsflores@ice.ufjf.br (L.S.F.); charcorrea@gmail.com (C.C.C.)

3 Centro Politécnico, Departamento de Química, Universidade Federal do Paraná, Curitiba 81530-900, PR, Brazil; tatianarenat@gmail.com

4 Instituto de Física, Universidade Federal de Goiás, Campus Samambaia, Goiânia 74690-900, GO, Brazil; anakarol213.quimica@gmail.com (A.K.S.M.V.); felipeterramartins@gmail.com (F.T.M.)

5 Centro Brasileiro de Pesquisas Físicas (CBPF), Urca 22290-180, RJ, Brazil; flavio.cbpf@gmail.com

6 Instituto de Química, Universidade Federal Fluminense, Niterói 24020-141, RJ, Brazil; guilherme_guedes@id.uff.br

7 Centro Federal de Educação Tecnológica de Minas Gerais, Departamento de Química, Belo Horizonte 30421-169, MG, Brazil; brenogalvao@gmail.com (B.R.L.G.); justinodanielle@gmail.com (D.D.J.); pauloortega@cefetmg.br (P.F.R.O.); wdpufmg@gmail.com (W.D.d.P.)

8 Department of Chemistry, Faculty of Philosophy Sciences and Letters of Ribeirao Preto, University of Sao Paulo, Ribeirão Preto 14040-901, SP, Brazil; jucancino@usp.br

9 Brazilian Synchrotron Light Laboratory (LNLS), Brazilian Center for Research in Energy and Materials (CNPEM), Campinas 13083-970, SP, Brazil; ricardo.reis@lnls.br

10 Departamento de Química—ICEx, Universidade Federal de Minas Gerais, Campus Pampulha, Belo Horizonte 31270-901, MG, Brazil; stumpf@ufmg.br

11 Departament de Química Inorgànica-Instituto de Ciencia Molecular (ICMol), Universitat de València, C/Catedrático José Beltrán 2, Paterna, 46980 València, Spain

* Correspondence: miguel.julve@uv.es (M.J.); maria.marinho@unifal-mg.edu.br (M.V.M.)

Chart 1: View of H_4mpba-based compounds since the first publication in 2001. Legend: the flags represent the number of published compounds with transition metal ions whereas the stars indicate the number of published compounds containing only Ni^{II} ions in each year. More details in Table S1.

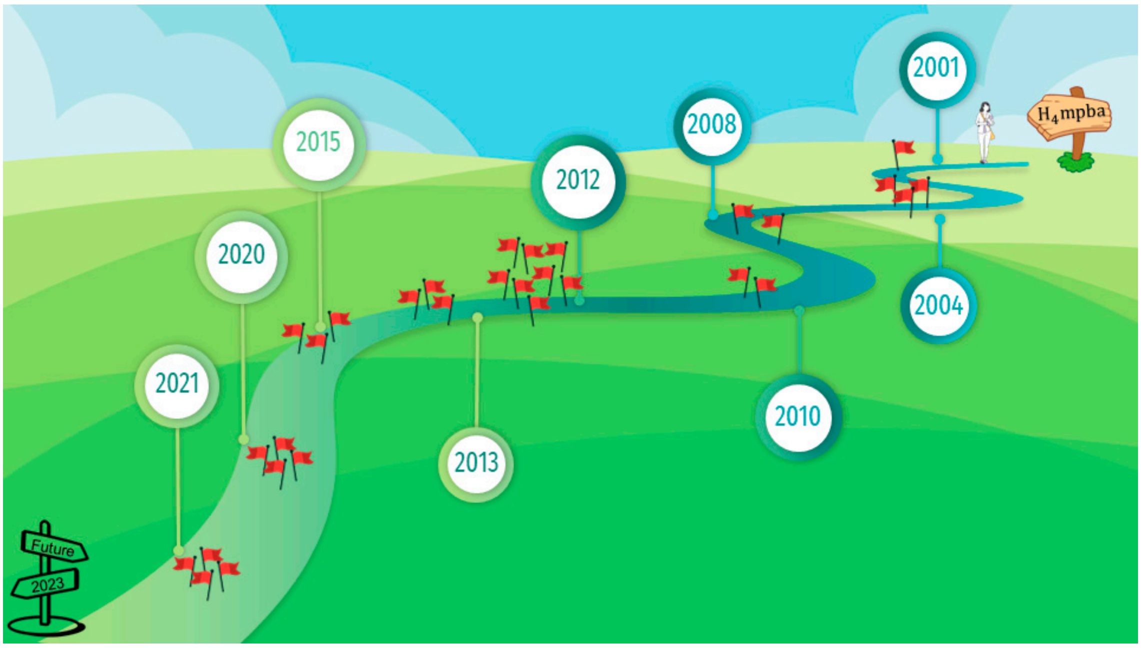

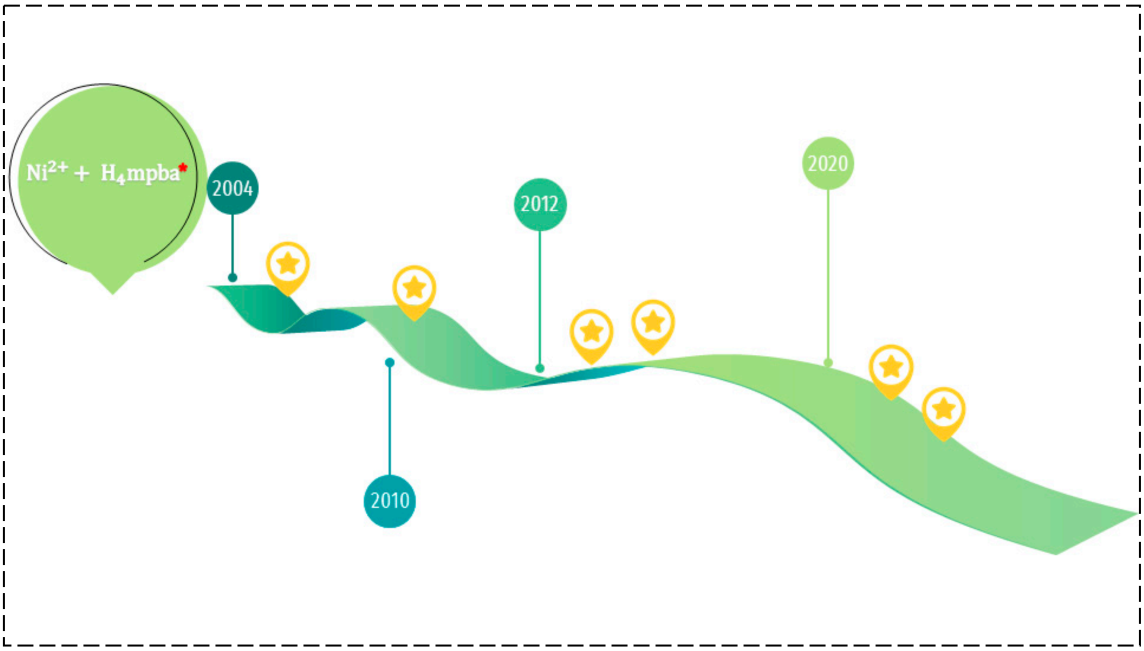

Table S1. Compounds containing the di-deprotonated (H_2mpba^{2-}) and fully deprotonated ($mpba^{4-}$) forms of the 1,3-phenylenebis(oxamic) acid (H_4mpba).

Molecular formula	Database identifier	Year	Reference
$Na_4[Cu_2(mpba)_2]\cdot 10H_2O$	VODXUH	2001	1
$[Co_2Cu_2(mpba)_2(H_2O)_6]\cdot 6H_2O$	-	2004	1
$Na_8[Ni_2(mpba)_3]\cdot 10H_2O$	FAJFIG	2004	2
$\{[Cu_2(mpba)_2][Cu(tmen)]_4\}(ClO_4)_4\cdot 6H_2O$	IYAVUZ	2004	3
$Li_5[Li_3Co_2(mpba)_3(H_2O)_6]\cdot 31H_2O$	POBNEA	2008	4
$Li_2[Mn_3Co_2(mpba)_3(H_2O)_6]\cdot 22H_2O$	-	2008	4
$Na_2\{[Cu_2(mpba)_3[Cu(Me_5dien)]_6\}(ClO_4)_6\cdot 12H_2O$	-	2010	5
$Na_2\{[Ni_2(mpba)_3][Cu(Me_5dien)]_6\}(ClO_4)_6\cdot 12H_2O$	-	2010	5
$[Na(H_2O)_4]_4[Mn_4\{Cu_2(mpba)_2(H_2O)_4\}3]\cdot 56\cdot 5H_2O$	DAPXID	2012	6
$Na_8[Ni^{II}_2(mpba)_3]\cdot 15H_2O$	VEHNED	2012	7
$Na_8[Co^{II}_2(mpba)_3]\cdot 17H_2O$	VEHNIH	2012	7
$TBA_2[Co_2(H_2mpba)_3]\cdot 2DMF\cdot 5H_2O$	KEVKON	2012	8
$(HNEt_3)_2[Co_2(H_2mpba)_3]\cdot 6DMF\cdot 5H_2O$	KEVKUT	2012	8
$TBA_2[Ni_2(H_2mpba)_3]\cdot 2DMF\cdot 2.5H_2O$	-	2012	8
$[Mn_4(H_2mpba)_4(H_2O)_{12}]\{[Mn_8Cu_8(mpba)_8(H_2O)_{24}]\}\cdot 29.5 H_2O$	QEJSEF	2012	9
$[Mn_4Cu_4(mpba)_4(H_2O)_9]\cdot 14H_2O$	QEJSIJ	2012	9
(S)-(1-Ph_Et)$Me_3N]_2[Mn_2(ox)Cu_2(mpba)_2(H_2O)_2]\}\cdot 3H_2O$	IDORAW	2013	10
$[Cu(bipy)(H_2mpba)]_2\cdot 2H_2O$	-	2013	10
$[Cu(bipy)(H_2mpba)]\cdot$dmso	-	2013	10
$K_4[Pd_2(mpba)_2]\cdot 4H_2O$	BUBGIQ	2015	11
$\{[K_4(H_2O)(dmso)][Pd_2(mpba)_2]\}$	BUBHIR	2015	11
$\{[Cu(bpca)]_4[Pd_2(mpba)_2]\cdot 6H_2O$	BUBHOX	2015	11
$\{[K_2(dmf)_2(H_2O)_2][Co_2(H_2mpba)_3]\cdot 2H_2O\}_n$	GUDHIZ	2020	12
$\{[K_2(dmf)_2(H_2O)_2][Ni_2(H_2mpba)_3]\cdot 2H_2O\}_n$	GUDHOF	2020	12
$[Co(tppz)_2][Co_2(H_2mpba)_3]\cdot 9H_2O$	GUDHUL	2020	12
$[Ni(tppz)_2][Ni_2(H_2mpba)_3]\cdot 9H_2O$	GUDJAT	2020	12
$[Co(H_2O)_6][Co_2(H_2mpba)_3]\cdot 2H_2O\cdot 0.5$dmso	UYIQEB	2021	13
$[Co(H_2O)_6][Co_2(H_2mpba)_3]\cdot 3H_2O\cdot 0.5$dpss	UYIQIF	2021	13
$[Co_2(H_2mpba)_2(H_2O)_4]_n\cdot 4nH_2O$	UYIQUR	2021	13
$[Co_2(H_2mpba)_2(CH_3OH)_2(H_2O)_2]_n\cdot 0.5nH_2O\cdot 2n$dpss	UYIQOL	2021	13

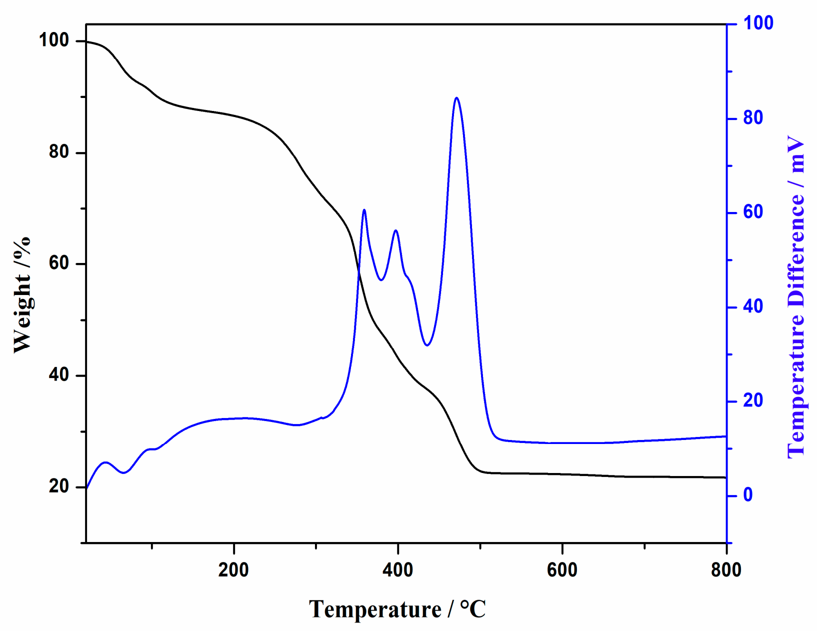

(**a**)

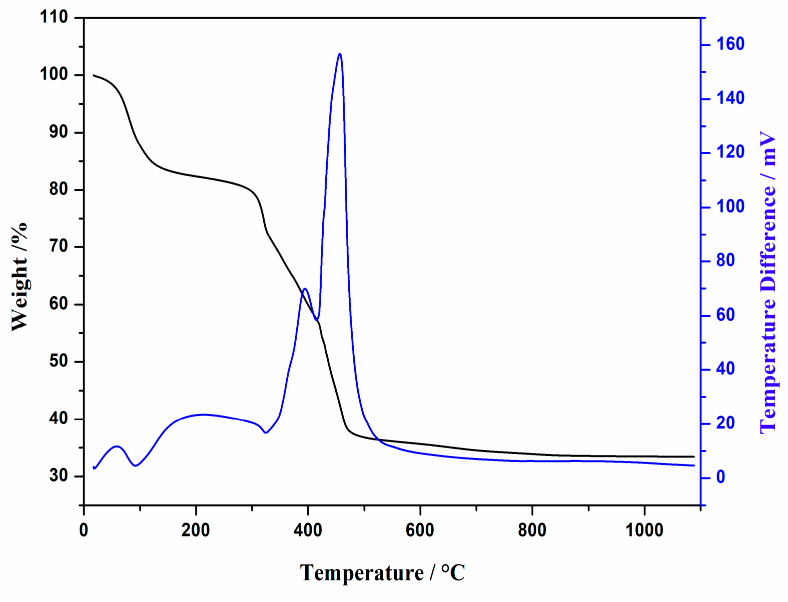

(**b**)

Figure S1. TG and DTA curves for (**a**) **1** and (**b**) **2**.

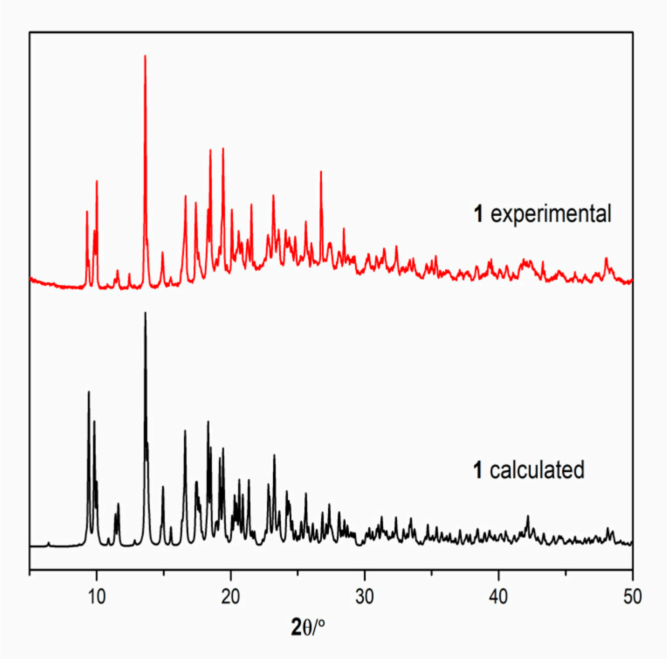

(**a**)

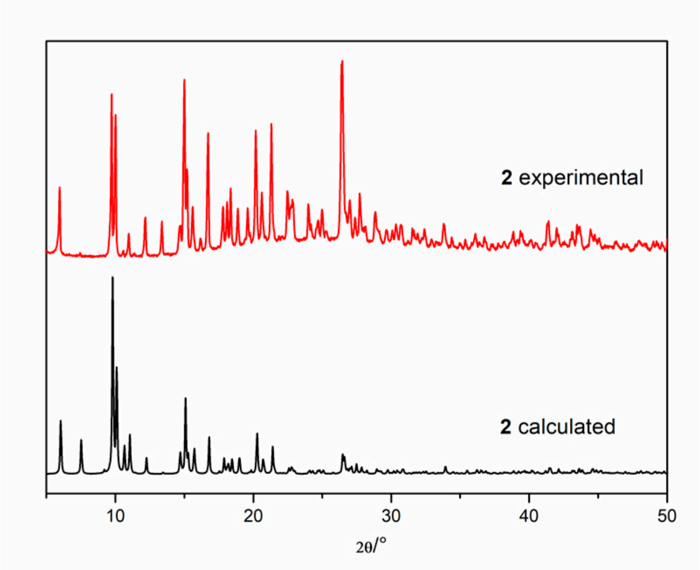

(**b**)

Figure S2. Experimental (red) and calculated (black) PXRD patterns of (a) **1** and (b) **2**.

Details for the crystallographic description of 1.

The H_2mpba^{2-} ligands are coordinated in three different coordination modes (Figure S3): i) one of them adopts the μ_4-κ^2O^7,O^8 (Ni1):κO^9 (1):κ^2O^{10},O^{11}(Ni2):κO^{11} (K1^{iv}) coordination mode. Only the N1 and N2 amide-nitrogen atoms act as hydrogen bond acceptors in this ligand, being involved in the N4-H4A···O19^{i} (coordinated dmso) and N3-H3A···O27 (free water molecule) type interactions. Otherwise, the hydrogen bond donor atoms concern the coordinated water molecules through O24^{v}-H24A^{v}···O12 and O25^{iv}-H25A^{iv}···O12 contacts. A very weak C22^{v}-H22^{v}···O7 type interaction also occurs; ii) Another one exhibits a μ_5-κ^2O^{16},O^{17} (Ni1):κ^2O^{17}, O^{18} (K2^{iii}):κO^{18} (K2):κ^2O^{13},O^{14} (Ni2):κO^{14} (K1^{iv}) coordination mode and it also forms hydrogen bonds [N5-H5···O26 (free water), N6-

H6A···O20^{viii} (lattice dmso) and O26-H26A···O15(oxamate) and weaker van der Waals contacts [C22-H22···O7^{vii} (oxamate), C2^{vi}-H2^{vi}···O15 (oxamate) and C33-H33A···O14 (oxamate)]; and iii) the third H_2mpba^{2-} ligand exhibits a μ_4-κ^2O^4,O^5 (Ni1): κ^2O^1,O^2 (Ni2):κO^2 (K1^{iv}):κO^3 (K1^{viii}) coordination mode. It is also involved in two hydrogen bonds [N1-H1···O26^{ix} and N2-H2A···O27^{ix} (free water molecule)] and a weaker van der Waals contact [C2-H2···O15^{ix} (oxamate)].

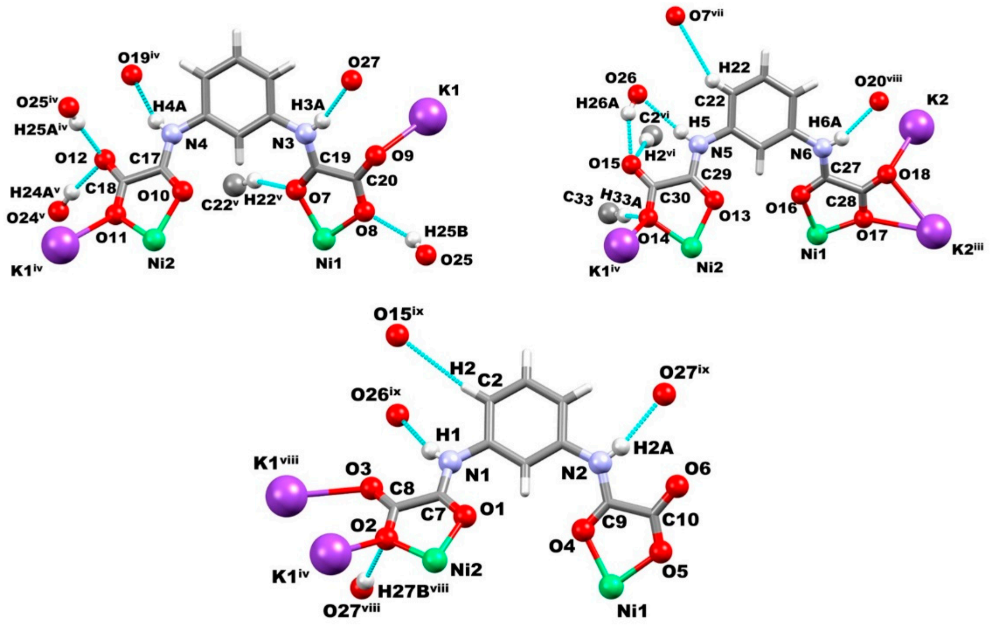

Figure S3. Coordination modes and hydrogen bonds for the H_2mpba^{2-} ligands in the helicate unit. Symmetry code : (iv) = *−x+3/2, y+1/2, −z+1/2*; (v) = *x+1/2,-y+1/2, z+1/2*; (vi) = *x-1, y, z*; (vii) = *x-1/2, -y+1/2, z-1/2*; (viii) = *x+1/2, -y+1/2, z-1/2*; (ix) = *x+1, y, z;* (x) = *-x+2, -y+1, -z*].

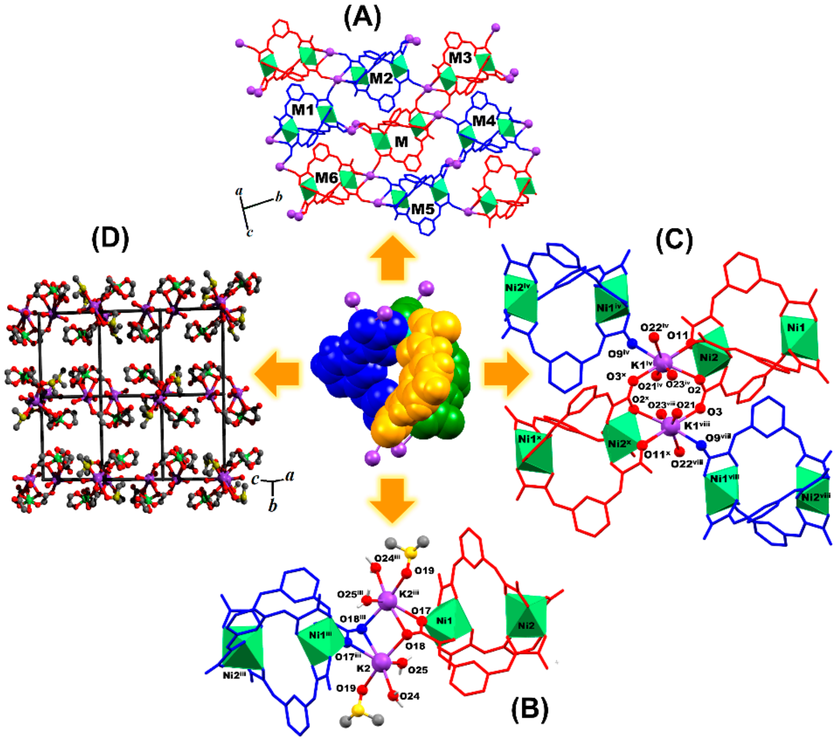

Figure S4. Topological features of the $[Ni_2(H_2mpba)_3]^{2-}$ helicate as a molecular building block (MBB) of the Ni^{II}/K^{I} heterometallic network in **1**. (A) Helicate neighbouring distribution (the **M** helicate as a central unit) in a 2D network along the diagonal of the *ac* plane; the red and blue-capped sticks represent the $[Ni_2(H_2mpba)_3]^{2-}$ helicate, the purple spheres concern the K^{I} ions and the green octahedra stand for the Ni^{II} ions. (B) H_2O/dmso-solvated K^{I}-dinuclear unit, drawn as a central unit and connecting two helicates. (C) Hydrated K^{I}-dinuclear unit, drawn as a central motif and connecting four distinct helicates. (D) Underlying net exhibiting the potassium oxo-metallic disposition in a **sql** topology. Overlap between the 2D underlying net (grid black sticks) with **sql** topology, and the two types of K^{I}-dinuclear units occupying the vertices of the 4,4-connected net [Symmetry code: (i) = -*x* + 3/2, *y* - 1/2, -*z* + 1/2; (ii) = *x* - 1/2, -*y* + 1/2, *z* + 1/2; (iii) = -*x* + 2, -*y*, -*z*; (iv) = −*x* + 3/2, *y* + 1/2, −*z* + 1/2; (v) = *x* + 1/2, -*y* + 1/2, *z* + 1/2; (vi) = *x* - 1, *y*, *z*; (vii) = *x* - 1/2, -*y* + 1/2, *z* - 1/2; (viii) = *x* + 1/2, -*y* + 1/2, *z* - 1/2, (ix) = *x* + 1, *y*, *z*; (x) = -*x* +2, -*y* +1, -*z*].

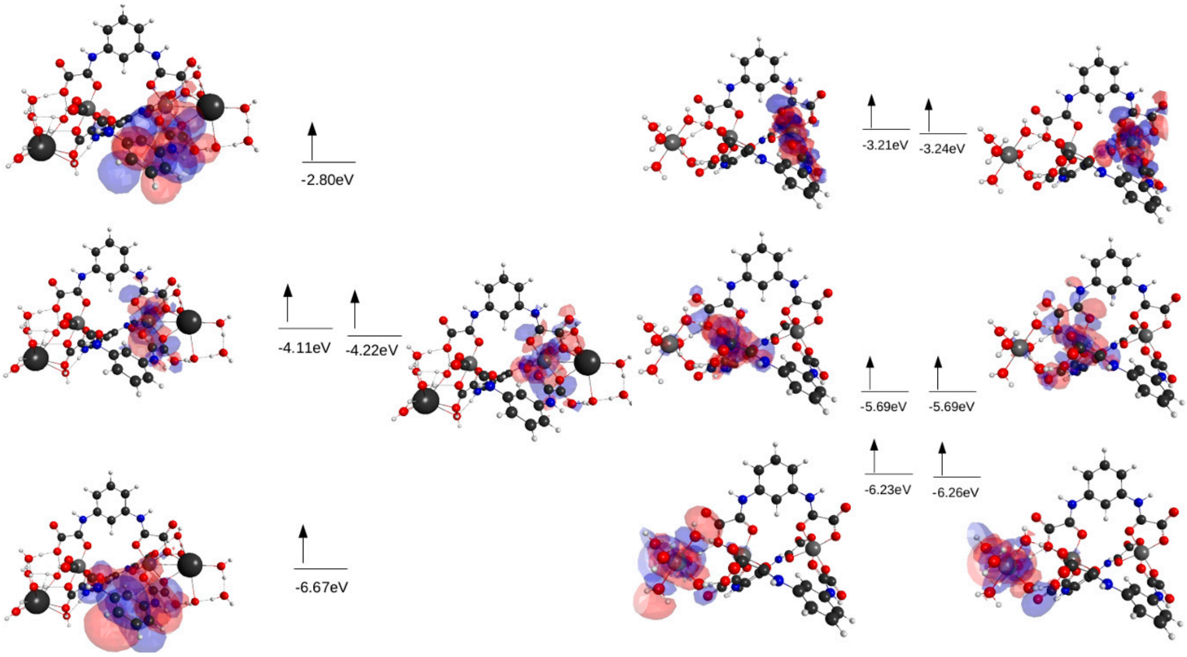

Figure S5. Frontier molecular orbitals calculated at the ωB97X-D/SBKJC level for **1** (left) and **2** (right).

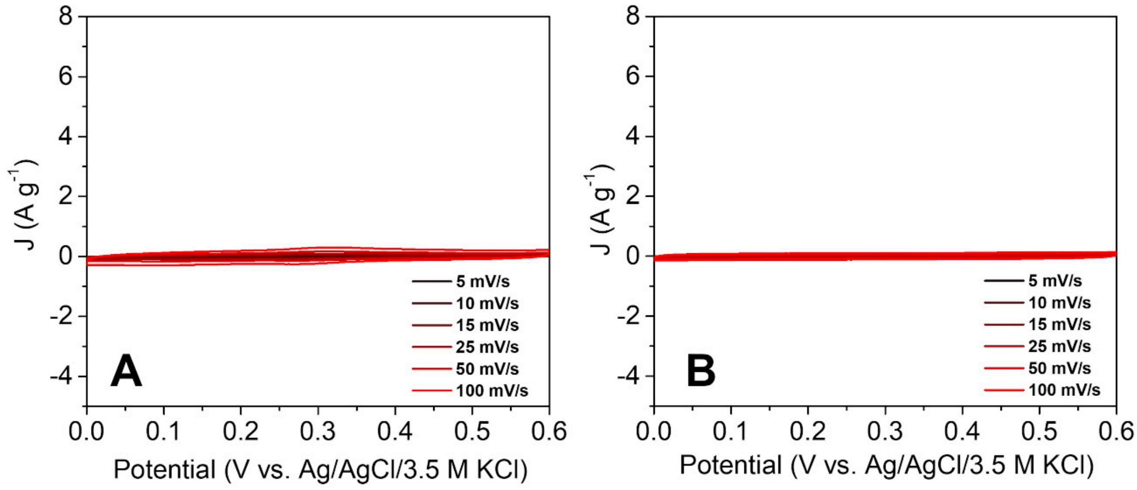

Figure S6. Cyclic voltammograms obtained at different scan rates (5 – 100 mV s^{-1}) for (A) **1** and (B) **2**. Electrolyte: 1.0 M KCl.

Table S2. Selected bond distances, bond angles, hydrogen bonds, and C-H···O type contacts in **1***.

Geometric parameters for **1**					
Bond distances (Å)					
O4-Ni1	2.059 (3)	O1-Ni2	2.054 (3)	O18-K2	2.738 (3)
O5-Ni1	2.033 (3)	O2-Ni2	2.035 (3)	O19-K2	2.682 (4)
O7-Ni1	2.064 (3)	O10-Ni2	2.064 (2)	O24-K2	2.856 (8)
O8-Ni1	2.025 (3)	O11-Ni2	2.022 (3)	O25-K2	2.662 (4)
O16-Ni1	2.059 (3)	O13-Ni2	2.062 (3)	O17^{iii}-K2	2.849 (3)
O17-Ni1	2.028 (3)	O14-Ni2	2.035 (3)	O18^{iii}-K2	2.904 (4)
O9-K1	2.760 (3)	O23-K1	3.042 (8)	O11^{i}-K1	3.290 (3)
O21-K1	2.756 (6)	O2^{i}-K1	2.918 (3)	O14^{i}-K1	2.789 (3)
O22-K1	2.962 (5)	O3^{ii}-K1	2.780 (3)	O18-K2	2.738 (3)
O19-K2	2.682 (4)	O24-K2	2.856 (8)	O25-K2	2.662 (4)
Bond angles (°)					
O8-Ni1-O17	93.23 (11)	O5-Ni1-O16	175.81 (12)	O8-Ni1-O7	81.26 (10)
O8-Ni1-O5	91.99 (12)	O8-Ni1-O4	171.99 (12)	O17-Ni1-O7	170.66 (13)
O17-Ni1-O5	95.82 (12)	O17-Ni1-O4	91.47 (12)	O5-Ni1-O7	91.93 (12)
O8-Ni1-O16	90.57 (12)	O5-Ni1-O4	81.08 (11)	O16-Ni1-O7	91.75 (11)
O17-Ni1-O16	80.72 (12)	O16-Ni1-O4	96.58 (11)	O4-Ni1-O7	94.89 (10)
O11-Ni2-O14	90.88 (12)	O2-Ni2-O1	81.02 (10)	O11-Ni2-O10	81.14 (11)
O11-Ni2-O2	94.10 (11)	O11-Ni2-O13	169.26 (12)	O14-Ni2-O10	90.07 (11)
O14-Ni2-O2	92.96 (11)	O14-Ni2-O13	80.97 (11)	O2-Ni2-O10	174.40 (11)
O11-Ni2-O1	91.93 (12)	O2-Ni2-O13	93.31 (11)	O1-Ni2-O10	96.12 (10)
O14-Ni2-O1	173.53 (11)	O1-Ni2-O13	96.91 (11)	O13-Ni2-O10	91.82 (10)
O21-K1-O9	69.19 (14)	O21-K1-O3^{ii}	100.85 (15)	O9-K1-O2^{i}	139.97 (10)
O21-K1-O22	152.20 (17)	O9-K1-O3^{ii}	81.05 (9)	O3^{ii}-K1-O2^{i}	127.07 (9)
O9-K1-O22	83.01 (13)	O21-K1-O14^{i}	120.26 (14)	O14^{i}-K1-O2^{i}	62.24 (8)
O21-K1-O23	144.37 (18)	O9-K1-O14^{i}	118.48 (9)	O3^{ii}-K1-O22	74.18 (12)

O9-K1-O23	133.30 (16)	O3^{ii}-K1-O14^{i}	138.24 (11)	O14^{i}-K1-O22	72.46 (11)
O22-K1-O23	58.75 (16)	O21-K1-O2^{i}	77.01 (13)	O2^{i}-K1-O22	128.23 (12)
O3^{ii}-K1-O23	64.42 (14)	O14^{i}-K1-O23	77.01 (13)	O2^{i}-K1-O23	86.73 (15)
O21-K1-O11^{i}	65.53 (14)	O9-K1-O11^{i}	89.22 (9)	O3^{ii}-K1-O11^{i}	165.57 (10)
O14^{i}-K1-O11^{i}	56.05 (8)	O2^{i}-K1-O11^{i}	56.77 (7)	O22-K1-O11^{i}	115.36 (11)
O25-K2-O19	80.68 (12)	O18-K2-O24	103.52 (17)	O19-K2-O18^{i}	98.96 (10)
O25-K2-O18	83.33 (11)	O25-K2-O17^{i}	130.90 (16)	O18-K2-O18^{i}	80.91 (10)
O19-K2-O18	164.00 (11)	O19-K2-O17^{i}	92.87 (10)	O17^{i}-K2-O18^{i}	45.34 (9)
O25-K2-O24	106.0 (2)	O18-K2-O17^{i}	98.12 (10)	O24-K2-O18^{i}	166.23 (18)
O19-K2-O24	80.40 (16)	O25-K2-O18^{i}	87.35 (16)		

Hydrogen bonds and C-H···O type contacts			
D-H···A	*d*(D···A)/Å	*d*(H···A)/Å	<(D-H···A)/°
N4-H4A···O19^{i}	2.908 (5)	2.090	158.7
N3-H3A···O27	3.032 (5)	2.908 (5)	164.6
O25^{iv}-H25A^{iv}···O12	2.927 (6)	2.08 (5)	171 (5)
O24^{v}-H24A^{v}···O12	2.952 (8)	1.966	170.9
O25-H25B···O8	2.852 (6)	2.06 (5)	152 (5)
C22^{v}-H22^{v}···O7	3.831 (5)	2.984	152.1
N5-H5···O26	3.085 (6)	2.229	173.1
N6-H6A···O20^{viii}	2.934 (6)	2.103	162.4
C22-H22···O7^{vii}	3.831 (5)	2.984	152.1
O26-H26A···O15	2.799 (6)	2.01 (6)	151 (5)
C2^{vi}-H2^{vi}···O15	3.723 (6)	2.800	171.7
C33-H33A···O14	3.808 (9)	2.890	160.4
N2-H2A···O27^{ix}	3.057 (5)	2.223	163.5
N1-H1···O26^{ix}	2.958 (6)	2.128	161.8
C2-H2···O15^{ix}	3.723 (6)	2.800	171.7

O26^{ix}-H26A^{ix}···O15^{ix}	2.799 (6)	2.01 (6)	151 (5)
O27^{viii}-H27B^{viii}···O2	3.122 (4)	2.35 (4)	156 (5)

* Symmetry code: (i) = -*x* + 3/2, *y* - 1/2, -*z* + 1/2; (ii) = *x* - 1/2, -*y* + 1/2, *z* + 1/2; (iii) = -*x* + 2, -*y*, -*z*; (iv) = −*x* + 3/2, *y* + 1/2, −*z* + 1/2; (v) = *x* + 1/2, -*y* + 1/2, *z* + 1/2; (vi) = *x* - 1, *y*, *z*; (vii) = *x* - 1/2, -*y* + 1/2, *z* - 1/2; (viii) = *x* + 1/2, -*y* + 1/2, *z* - 1/2; (ix) = *x* + 1, *y*, *z*; (x) = -*x* + 2, -*y* + 1, -*z*.

Table S3. Selected bond lengths, bond angles, and hydrogen bonds in **2 *.**

Geometric parameters for **2**					
Bond distances (Å)					
Ni1-O1	2.033 (2)	Ni1-O7	2.047 (3)	Ni1-O5	2.051 (2)
Ni1-O2	2.078 (2)	Ni1-O8	2.054 (2)	Ni1-O4	2.045 (2)
Ni2-O12	2.026 (3)	Ni2-O10	2.070 (3)	Ni2-O11^{ii}	2.053 (3)
Ni2-O11	2.053 (3)	Ni2-O12^{ii}	2.026 (3)	Ni2-O10^{ii}	2.070(3)
Bond angles (°)					
O1-Ni1-O2	80.30 (9)	O8-Ni1-O2	92.81 (9)	O4-Ni1-O5	80.70 (9)
O1-Ni1-O8	170.70 (10)	O5-Ni1-O2	171.76 (10)	O4-Ni1-O7	170.98 (10)
O1-Ni1-O5	93.06 (9)	O5-Ni1-O8	94.31 (10)	O7-Ni1-O2	94.45 (10)
O1-Ni1-O4	90.04 (10)	O4-Ni1-O2	94.36 (9)	O7-Ni1-O8	80.86 (10)
O1-Ni1-O7	93.37 (11)	O4-Ni1-O8	96.76 (9)	O7-Ni1-O5	90.77 (10)
O12-Ni2-O11	92.68 (13)	O12^{i}-Ni2-O11^{i}	92.68 (13)	O11-Ni2-O10^{i}	89.45 (14)
O11-Ni2-O10	90.55 (14)	O12^{i}-Ni2-O10^{i}	92.14 (14)	O11^{i}-Ni2-O10	89.44 (14)
O12^{i}-Ni2-O12	180.0	O12^{i}-Ni2-O10	87.86 (14)	O11^{i}-Ni2-O10^{i}	90.55 (14)
O12^{i}-Ni2-O11	87.32 (13)	O12-Ni2-O10^{i}	87.86 (14)	O10^{i}-Ni2-O10	180.0
O12-Ni2-O11^{i}	87.32 (13)	O11-Ni2-O11^{ii}	180.0	O12-Ni2-O10^{i}	87.86 (14)

Hydrogen bonds

D-H···A	*d*(D···A)/Å	*d*(H···A)/Å	<(D-H···A)/°
N1-H1···O3^{iii}	2.075	2.882 (4)	155.9
N2-H2···O6^{iv}	2.144	2.972 (4)	161.3
O12-H12B···O1	1.878	2.684 (4)	157.5
O10-H10B···O5	1.898	2.718 (4)	161.7

O11-H11B···O7	2.009	2.801 (4)	154.3

* Symmetry code: (i) = -*x*, -*y*, -*z*; (ii) = -*x* + 5/4, -*y* + 1/4, *z*; (iii) = -*x* + 7/4, *y*, -*z* + 7/4; (iv) = *x*, -*y* + 1/4, -*z* + 9/4.

Table S4. Possible geometries of eight-coordinated metal centers and deviation parameters from each ideal polyhedron for Ni and K in **1**.

Geometry	Point group	Shape	K1
OP-8	D8h	Octagon	24.559
HPY-8	C7v	Heptagonal pyramid	20.885
HBPY-8	D6h	Hexagonal bipyramid	21.459
CU-8	Oh	Cube	21.227
SAPR-8	D4d	Square antiprism	11.739
TDD-8	D2d	Triangular dodecahedron	11.249
JGBF-8	D2d	Johnson gyrobifastigium J26	15.723
JETBPY-8	D3h	Johnson elongated triangular bipyramid J14	15.973
JBTPR-8	C2v	Biaugmented trigonal prism J50	8.731
BTPR-8	C2v	Biaugmented trigonal prism	11.084
JSD-8	D2d	Snub disphenoid J84	6.921
TT-8	Td	Triakis tetrahedron	21.700
ETBPY-8	D3h	Elongated trigonal bipyramid	20.234

Table S5. Possible geometries of eight-coordinated metal centers and deviation parameters from each ideal polyhedron for Ni in **2**.

Geometry	Point group	Shape	K2	Ni1	Ni2
HP-6	D6h	Hexagon	28.392	12.880	13.126
PPY-6	C5v	Pentagonal pyramid	22.555	23.457	23.717
OC-6	Oh	Octahedron	18.969	11.072	11.220
TPR-6	D3h	Trigonal prism	21.598	17.800	18.905
JPPY-6	C5v	Johnson pentagonal pyramid J2	22.324	24.119	23.739

Geometry	Point group	Shape	Ni1	N12
HP-6	D6h	Hexagon	24.245	24.047
PPY-6	C5v	Pentagonal pyramid	23.308	27.531
OC-6	Oh	Octahedron	7.411	7.746
TPR-6	D3h	Trigonal prism	15.237	18.086
JPPY-6	C5v	Johnson pentagonal pyramid J2	24.661	29.041

References

1. Ruiz, R.; Faus, J.; Julve, M.; Lloret, F.; Cano, J.; Ottenwaelder, X.; Journaux, Y.; Mun, M.C. Ferromagnetic Coupling through Spin Polarization in a Dinuclear Copper(II) Metallacyclophane. *Angew. Chem. Int. Ed.* **2001**, *1*, 3039–3042.
2. Pardo, E.; Morales-Osorio, I.; Julve, M.; Lloret, F.; Cano, J.; Ruiz-García, R.; Pasán, J.; Ruiz-Pérez, C.; Ottenwaelder, X.; Journaux, Y. Magnetic Anisotropy of a High-Spin Octanuclear Nickel(II) Complex with a Meso-Helicate Core. *Inorg. Chem.* **2004**, *43*, 7594–7596.
3. Pardo, E.; Bernot, K.; Julve, M.; Lloret, F.; Cano, J.; Ruiz-García, R.; Delgado, F.S.; Ruiz-Pérez, C.; Ottenwaelder, X.; Journaux, Y. Spin Control in Ladderlike Hexanuclear Copper(II) Complexes with Metallacyclophane Cores. *Inorg. Chem* **2004**, *43*, 2768–2770.
4. Pardo, E.; Cangussu, D.; Dul, M.-C.; Lescouëzec, R.; Herson, P.; Journaux, Y.; Pedroso, E.F.; Pereira, C.L.M.; Muñoz, M.C.; Ruiz-García, R.; Cano, J.; Amorós, P.; Julve, M.; Lloret, F. A Metallacryptand-Based Manganese(II)–Cobalt(II) Ferrimagnet with a Three-Dimensional Honeycomb Open-Framework Architecture. *Angew. Chem. Int. Ed.* **2008**, *47*, 4211–4216.

5. Hill, S.; Datta, S.; Liu, J.; Inglis, R.; Milios, C.J.; Feng, P.L.; Henderson, J.J.; del Barco, E.; Brechin, E.K.; Hendrickson, D.N. Magnetic Quantum Tunneling: Insights from Simple Molecule-Based Magnets. *Dalton Trans.* **2010**, *39*, 4693–4707.
6. Ferrando-Soria, J.; Ruiz-García, R.; Cano, J.; Stiriba, S.E.; Vallejo, J.; Castro, I.; Julve, M.; Lloret, F.; Amorõs, P.; Pasán, J.; Ruiz-Pérez, C.; Journaux, Y.; Pardo, E. Reversible Solvatomagnetic Switching in a Spongelike Manganese(II)- Copper(II) 3D Open Framework with a Pillared Square/Octagonal Layer Architecture. *Chem. Eur. J.* **2012**, *18*, 1608–1617.
7. Dul, M.C.; Lescouëzec, R.; Chamoreau, L.M.; Journaux, Y.; Carrasco, R.; Castellano, M.; Ruiz-García, R.; Cano, J.; Lloret, F.; Julve, M.; Ruiz-Pérez, C.; Fabelo, O.; Pardo, E. Self-Assembly, Metal Binding Ability, and Magnetic Properties of Dinickel(II) and Dicobalt(II) Triple Mesocates. *CrystEngComm.* **2012**, *14*, 5639–5648.
8. Lisnard, L.; Chamoreau, L.M.; Li, Y.; Journaux, Y. Solvothermal Synthesis of Oxamate-Based Helicate: Temperature Dependence of the Hydrogen Bond Structuring in the Solid. *Cryst. Growth Des.* **2012**, *12*, 4955–4962.
9. Ferrando-Soria, J.; Grancha, T.; Pasán, J.; Ruiz-Pérez, C.; Cañadillas-Delgado, L.; Journaux, Y.; Julve, M.; Cano, J.; Lloret, F.; Pardo, E. Solid-State Aggregation of Metallacyclophane-Based $Mn^{II}Cu^{II}$ One-Dimensional Ladders. *Inorg. Chem.* **2012**, *2*, 7019–7021.
10. Grancha, T.; Tourbillon, C.; Julve, M.; Lloret, F.; Fabelo, O.; Pardo, E. Self-Assembly of a Chiral Three-Dimensional Manganese(II)–Copper(II) Coordination Polymer with a Double Helical Architecture. *CrystEngComm.* **2013**, *2*, 9312–9315.
11. Oliveira, W.X.C.; Ribeiro, M.A.; Pinheiro, C.B.; Da Costa, M.M.; Fontes, A.P.S.; Nunes, W.C.; Cangussu, D.; Julve, M.; Stumpf, H.O.; Pereira, C.L.M. Palladium(II)-Copper(II) Assembling with Bis(2-pyridylcarbonyl)amidate and Bis(oxamate) Type Ligands. *Cryst. Growth Des.* **2015**, *15*, 1325–1335.
12. Mariano, L.D.S.; Rosa, I.M.L.; de Campos, N.R.; Doriguetto, A.C.; Dias, D.F.; do Pim, W.D.; Valdo, A.K.S.M.; Martins, F.T.; Ribeiro, M.A.; de Paula, E.E.B.; Pedroso, E. F.; Stumpf, H. O.; Cano, J.; Lloret, F.; Julve, M.; Marinho, M.V. Polymorphic Derivatives of Ni^{II} and Co^{II} Mesocates with 3D Networks and "Brick and Mortar" Structures: Preparation, Structural Characterization, and Cryomagnetic Investigation of New Single-Molecule Magnets. *Cryst. Growth Des.* **2020**, *20*, 2462–2476.
13. De Campos, N.R.; Simosono, C.A.; Rosa, I.M.L.; Silva, R.M.R. da; Doriguetto, A.C.; Pim, W.D. do; Simões, T.R.G.; Valdo, A.K.S.M.; Martins, F.T.; Sarmiento, C. V.; Nunes, W. C.; Guedes, G. P.; Pedroso, E. F.; Pereira, C. L.M.; Stumpf, H.O., Lloret, F.; Julve, M.; Marinho, M.V. Building-up Host–Guest Helicate Motifs and Chains: A Magneto-Structural Study of New Field-Induced Cobalt-Based Single-Ion Magnets. *Dalton Trans.* **2021**, *50*, 10707–10728.
